# Psychological Flexibility and Mindfulness as Predictors of Individual Outcomes in Hospital Health Workers

**DOI:** 10.3389/fpsyg.2019.01302

**Published:** 2019-06-12

**Authors:** Tiziana Ramaci, Diego Bellini, Giovambattista Presti, Giuseppe Santisi

**Affiliations:** ^1^Faculty of Human and Social Sciences, University of Enna “Kore,” Enna, Italy; ^2^Faculty of Law, University of Cagliari, Cagliari, Italy; ^3^Department of Educational Sciences, University of Catania, Catania, Italy

**Keywords:** occupational stress factors, mindfulness, attention, experiential avoidance, ACT, health

## Abstract

Research in organizational psychology emphasizes the idea that wellbeing and productivity outcomes are influenced both by individual differences (traits, values) and work environment characteristics (relationships, climate). Evidence on the effectiveness of psychological interventions for stress is currently unclear. To date, research on psychological flexibility in workplaces has not been systematically conducted in Italy. We investigated its relevance in the context of the Italian health care system. In this study, the relationship between sources of stress at work and its outcomes in terms of psychological and physical health are explored. Furthermore, the moderating effect of psychological flexibility and mindfulness on psychological and physical health are investigated. Four hundred and eleven health workers from a Sicilian hospital, with different job positions were recruited, of which 42.7% were males (*N* = 169) and 57.3% were females (*N* = 227). Their ages ranged between 25 and 72 years (*M* = 49.16; *SD* = 8.65). Participants answered a questionnaire that assessed psychological flexibility, mindfulness, sources of stress at work and health benefits. In a bivariate analysis, managerial factors (MF), relationships, and intrinsic factors are partially negatively related to psychological and physical health; whereas, multivariate analyses show that psychological flexibility does not moderate the relationship between psychological and physical health. Instead, mindfulness is strongly and consistently correlated to psychological and physical health. Employees who show psychological flexibility, are more likely to show greater openness to the acceptance of setbacks in the working environment and to carry on their valued living and working path. This seems to correlate positively on individual wellbeing. Data show that a flexible and mindful attitude toward difficult psychological events aids responsiveness to changes and the ability to work more effectively.

## Introduction

Psychological flexibility is defined as being in contact with the present moment, fully aware of emotions, sensations, and thoughts, welcoming them, including the undesired ones, and moving in a pattern of behavior in the service of chosen values. In simpler words this means accepting our own thoughts and emotions and acting on long-term values rather than short-term impulses, thoughts, and feelings that are often linked to experiential avoidance and a way to control unwanted inner events (Hülsheger et al., 2013). Acceptance and Commitment Therapy (ACT) is a transdiagnostic cognitive-behavioral intervention that aims to foster psychological flexibility as a central means to human adaptation and wellbeing (Biglan et al., 2008). In a non-clinical context, it can also be called Acceptance and Commitment Training.

There is good empirical evidence that targeting psychological flexibility with an ACT protocol is beneficial for a range of clinical disorders including anxiety, depression, and post-traumatic stress disorders (Powers et al., 2009), and with patients with chronic physical diseases (Prevedini et al., 2011). Broadly speaking such interventions have a general effect measured by a wide range of outcomes, including quality of life, stress, and benefit finding (Petralia et al., 2018). Studies also report improvements across a range of outcomes including self-management and coping, reduced distress and perceived stigma, and improved quality of life.

Research in healthcare settings has mostly focused on organizational aspects around workplace conflict and safety perception such as workload, relationship to management, colleagues, work position, and home. Studies have largely neglected to investigate the role of factors associated with Psychological Flexibility patterns, which have been related to adaptive responses to heavy stressors, both in terms of frequency and intensity, like those found in hospitals (Salminen et al., 2003; Rugulies et al., 2007; Paquet et al., 2013; Ramaci et al., 2016, 2017; Ledda et al., 2017; Magnano et al., 2017).

Occupational stress is a serious health issue affecting organizations and employees. Over the last few years in Italy, many changes have occurred in workplaces that led to organizations focusing more on work-related stress. The introduction of a specific law on safety in the workplace, Dlg. 81/2008 (recently updated in July 2018), made it mandatory for organizations to carry out work-related stress risk assessments, and implement, when necessary, actions aimed at preventing, reducing, and eliminating sources of risk (Paolillo et al., 2015)^1^.

Nowadays in all working contexts there is an increased awareness of the negative impact of stress on the physical, psychological, and social health of individuals (Specchiale et al., 2013). In the European Working Conditions Survey (EWCS 2000), work-related stress was found to be the second most common work-related health problem across the EU15 (at 28%; only back pain was more common)^2^.

Stress and Psychological Flexibility are two intertwined constructs. In Selye (1974) provided a still valid definition of stress considering it “a non-specific response of an individual to any request of change” (p. 233). According to the current psycho-physiological definition, stress is a notable and persistent condition in which an organism is exposed to risk factors, which tend to alter its balance or homeostasis. Therefore, it is intuitive that a flexible and adaptive repertoire is crucial in response to a stressful environment.

In the last decades, the idea of wellbeing counteracting work related stress has been linked not only to the individual employees’ characteristics (e.g., traits, personal values, etc.) but also the whole organization itself, conceptualized as a complete organic system. Organizational wellbeing is thus related to all organizational aspects including climate, culture, and performance.

In healthcare settings, contact with ailing patients and their relatives represents an emotionally complex situation which, if not properly managed, may in the long-term threaten the psychological health and quality of life of healthcare workers and consequently negatively impact their performance. In this context, healthcare workers’ inner states (thoughts, emotions, past experiences, and memories) are fundamental to how these stressors are handled. Attempts to avoid thoughts, feelings, and emotions that occur under various stressful conditions in hospital settings have been found to relate to stress, absenteeism, depression, and ineffective communication or interpersonal relationships, rather than a lack of technical skills (Monestès et al., 2016). In the ACT literature, this behavioral pattern comes under the term of experiential avoidance. It occurs when a person is unwilling to stay in contact with private experiences (e.g., bodily sensations, emotions, thoughts, memories, images, and behavioral predispositions) that are judged painful and tries to alter the form or frequency of these experiences or the situations that trigger them, even when behavioral harm or dysfunctional patterns of behavior are a consequence (Hayes et al., 2005).

An increasing number of studies on experiential avoidance and thought suppression point to the fact that mental health and everyday behavioral patterns of action are influenced more by the way people relate to their thoughts and feelings than by their actual form (e.g., how unpleasant their thoughts or feelings are) (Bond et al., 2006). For example, in a population suffering from chronic pain, disability is related more to experiential avoidance of pain than to the degree of pain itself (McCracken, 1998). In contrast, Psychological Flexibility is defined as the open acceptance of unpleasant sensations, thoughts, and feelings, while focusing on the present moment, which allows an individual to act according to the context required in the pursuit of one’s goals and values (Hayes et al., 1999; Bond and Flaxman, 2006). As a consequence, “psychologically flexible” individuals will show a reduced tendency to control internal events, when doing so prevents them from taking goal-directed action. This mindful non-judgmental stance allows people to focus more on the opportunities associated with the present moment, with positive effects on job performance, motivation, absenteeism, and mental health at work (Bond and Hayes, 2002), and less emotional disturbance (Baer, 2003; Hayes et al., 2006).

Psychological flexibility is founded on the six core ACT processes: defusion, acceptance, present moment, self-as-a-content, values, and committed action. ACT uses acceptance and mindfulness strategies to develop committed behavior change by increasing it. Where the established operational definition offered by Kabat-Zinn (2003) of mindfulness is “the awareness that emerges through paying attention on purpose, in the present moment, and non-judgmentally to the unfolding of experience moment by moment” (p. 145), in the ACT model this state of purposeful awareness is promoted using metaphors and experiential exercises (including meditation) by working on defusion, acceptance, present moment, and self as a context. So, mindfulness, as an awareness state, is only one component of the psychological flexibility construct, the other being commitment to a valued action life path.

On the other side of psychological flexibility there is inflexibility. Experiential avoidance is the process at the core of the empirically based conceptualization of psychopathology in the ACT model (Hayes et al., 1999; Monestès et al., 2016). In ACT terms, the most common “problematic” psychological aspects often find their origins in language processes such as the exertion of control by certain verbal processes, including covert ones. Ruiz and Odriozola-González (2017) demonstrated that general psychological flexibility and work-related psychological flexibility might moderate different areas of individuals’ adaptive skills. They found a stronger relationship between exhaustion and cynicism and psychological flexibility for individuals with low levels of work-related psychological flexibility compared to participants with high levels. People are more psychologically healthy and perform more effectively when they base their pattern of action, both at work or in other contexts, on their own values and goals. By modifying the verbal context in which problematic psychological events occur, a therapist can alter the function of covert events (thoughts) on behavior where an individual becomes more “sensitive” to personal contingencies of reinforcement present in the environment (Bellini et al., 2019). Acting in a psychologically flexible manner, therefore, means persisting with or modifying one’s behavior according to one’s most intrinsic motivations and personal values, making oneself available to experience when it happens in the present moment.

Within this theoretical framework and application in organizations, ACT (Hayes et al., 1999) is proving to be one of the most effective intervention models to weaken problematic psychological processes and to re-establish more functional ones. Several studies (see Bond et al., 2016 for a review) show that psychological flexibility is positively correlated with wellbeing and can predict health, attitudinal, and productivity outcomes in organizational contexts. Engaging with values related to contingencies, people increase their sense of effectiveness and improve their level of health. The results of a randomized study investigating the effect of an ACT-based protocol on work stress reduction, mental health status, and the development of a propensity for innovation (Bond and Bunce, 2003) showed that positive outcomes were mediated by an increase in psychological flexibility. The comparison between the experimental subjects, the control group, and a sample of individuals participating in a different training, but with the same objectives, showed that the results achieved by the ACT group were not determined by the contents of thoughts with respect to stressful work-related situations, but to the maturation of a functional perspective toward these thoughts.

Similar results were obtained by Bond and Flaxman (2006) who compared the effectiveness of two interventions aimed at promoting organizational wellbeing; one focused on a protocol based on ACT and the other on a more traditional Cognitive Behavioral Therapy (CBT) program based on Stress Inoculation Training. As hypothesized by the authors, both trainings showed positive results; however, they were mediated by different processes. In the first case, there was an increase in psychological flexibility (ACT protocol); in the second, there was an attenuation of negative contents of thoughts (CBT program).

Another interesting result that emerges from research data is the link between psychological flexibility and the construct of job control, defined as the perceived ability to exert influence over one’s work context to make it more rewarding and less threatening. Bond and Bunce (2003) identified a positive correlation between Psychological Flexibility and job control that correlated, in a follow-up survey, 1 year later, with better levels of mental health.

It is therefore important to analytically assess the conditions under which stress occurs both to guide the choice of the type of intervention to be implemented, but also to clarify to trainers and trainees the current situation and modifications that can be obtained. To accomplish this integrated assessment, numerous tools are available in literature (Harris, 2010; Pellerone et al., 2017a).

Different studies (Bond et al., 2008; Levin et al., 2012) have also shown that psychological flexibility correlates well with wellbeing within organizational contexts. By contacting the contingencies related to one’s own values, people increase their sense of effectiveness and improve their health by reducing negative performance outcomes. In general, the results of these studies highlight how psychological flexibility positively influences the wellbeing of individuals, making them more open to change and able to work effectively, which appears to reflect positively on psychological health and organizational wellbeing (Pellerone et al., 2017b).

Based on recent risk management theories, there is a need for specific plans aimed at identifying, analyzing, assessing, and constantly monitoring the possible risks of stress in the workplace, in order to prevent or avoid damaging effects. Risk management is a recursive process, where initial identification of risk factors is followed by periodic cycles of evaluation and interventions to progressively optimize organizational aspects that reduce risk and increase the wellbeing of employees (Santisi et al., 2018).

In general, the results of these studies within organizational contexts highlight how ACT-based interventions, which build psychological flexibility positively influence individuals and make them more aware and responsive to change and enable them to work more efficiently. More flexible workers are therefore able to notice, with greater openness, opportunities where they can express their valued pattern of behavior in the workplace and this seems to correlate positively on their individual wellbeing (Levinthal and Rerup, 2006). Thus, increasing an individual’s psychological flexibility in an organization has proved an excellent strategy to reduce stress and increase coping (Bond and Bunce, 2003; Biglan et al., 2008; Atkins and Parker, 2012).

**FIGURE 1:**
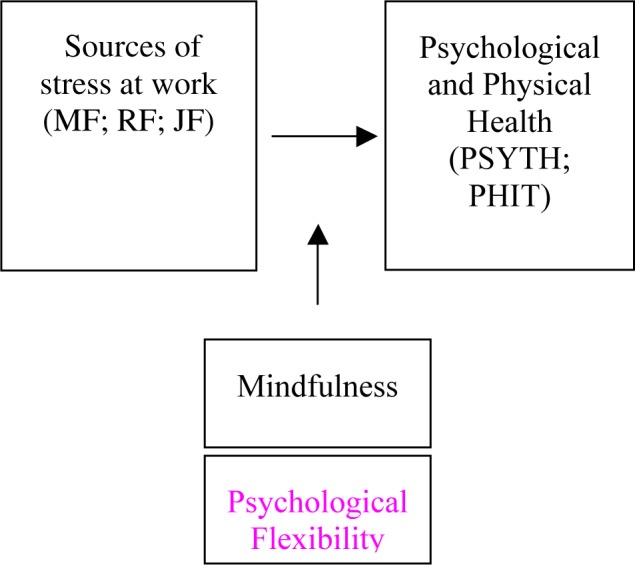
Conceptual model.

In our study, we aimed to investigate the relationship between sources of stress in the workplace, focusing more specifically on managerial factors (MF) such as both power and decision, relationships at work, intrinsic factors and, psychological and physical health. Furthermore, the moderating effect of flexibility (Bond and Flaxman, 2006) and mindfulness (Bond and Bunce, 2003; Butler and Gray, 2006) on symptoms of occupational ill health and its relationships (psychological and physical health) were analyzed. The conceptual model is depicted in Figure 1. These relationships have been studied within the occupational stress factors based on Cooper and Marshall’s (1976) original model of work-related stress, which has been described as measuring a number of work-related stressors and stress outcomes. The model comprises five sources of stress: intrinsic to the job, including poor physical working conditions, work overload; role in the organization, such as role ambiguity and role conflict; career development; relationships at work; organizational climate. These work stressors are relevant to individual and organizational experiencing negative stress outcomes.

## Study Aim and Hypotheses

The present study aimed to investigate how flexibility and mindfulness buffer the relationship between MF, both power and decision, relationships at work, intrinsic factors, and psychological and physical.

The following hypotheses were proposed:

Managerial factors, both decision (H1) and power (H2), relationships (RF: H3), and intrinsic factors (JF: H4) are negatively related to psychological and physical health.

Flexibility will moderate the relationship between MF (power: H5 and decision: H6), relationships (RF: H7), and intrinsic factors (JF: H8) on psychological and physical health.

Mindfulness will moderate the relationship between each independent variable: Managerial decision (H9), managerial power (H10), relationships at work (H11), and intrinsic factors (H12) on psychological and physical health.

## Materials and Methods

### Sample

In this study, qualified researchers interviewed 432 employees from a Sicilian hospital of the National Health System, who took part in the study on a voluntary basis. All data were managed according to the EU General Data Protection Regulation (GDPR) The Internal Review Board (IRB) of the Faculty of Human and Social Sciences at the “Kore” University of Enna approved the present research. The data were collected from February to December 2017. All participants were asked to complete a questionnaire, with 411 workers choosing to participate (95% response rate). Missing data were analyzed and outliers were screened in the dependent variables (psychological and physical health) to test research hypotheses for each record. No missing data were over 5%, no records were deleted according to this criterion (Graham, 2009). We standardized dependent variables and no *z* value was equal or over ± 3, then, according this criterion (Tabachnick and Fidell, 2013) no outliers were detected.

Respondents comprised 42.7% (*N* = 169) males and 57.3% (*N* = 227) females. Their average age was 49.16 years (*SD* = 8.65). Sixty-seven (19.5%) of them were physicians, 148 (43.1%) nurses, 44 (12.8%) administrative staff, three (0.9%) psychologists, 14 (4.1%) hospital staff, and 167 (19.5%) other health professions. Tenure average was 15.42 (*SD* = 8.63) years. The characteristics of the sample are reported in Table 1.

**Table 1:** Sample characteristics.

Variables	Results	
	Number of subj.	Percentage
Schooling		
High	129	33.6%
Graduate	255	27.5%
Area of expertise		
Administrative	25	6.6%
Healthcare	272	72.0%
Technical	81	21.4
Type of contract		
Long-term	340	87.6%
Fixed-term	48	12.4%
Time		
Full time	383	97.7%
Part time	9	2.3%
Work shift		
Yes, daily	130	34.3%
Yes, day and night	142	37.5%
No	107	28.2%
Work position Physician	67	19.5%
Nurse	148	43.1%
Other health care professions^∗^	67	19.5%
Psychologist	3	0.9%
Hospital Operators	14	4.1%
Administrative	44	12.8%
Hours of service (daily)	Mean = 6.98; *SD* = 1.33	
Years of seniority	Mean = 15.42; *SD* = 8.63	

*^∗^These include obstetricians, rehabilitation operators, technical lab. operators, and prevention technicians.*

Although the sample is strongly weighted toward nurses who comprise 43.1% of the sample (compared to 19.5% physicians and 19.5% other health professions), it reflects the distribution of the hospital workforce with around 48% being nurses (*N* = 650) versus 27% (*N* = 370) being physicians.

### Measures

This study was conducted using the following questionnaires:

OSI – The Occupational Stress Indicator [Cooper et al., 1988a,b; Italian version by Sirigatti and Stefanile (2002)] is a test designed to detect a broad spectrum of psychosocial stress, within an organization, classified under four areas: sources of stress, individual characteristics, coping, and consequences of stress). A total of 167 items are rated on a 6-point response scale, ranging from 1 (absolutely false) to 6 (absolutely true). For the purposes of this study only two areas were investigated:

• Sources of stress – sub scale: Managerial factors both decision and power (MF: 11 items), relationships at work (RF: 10 items) and intrinsic factors (JF: 9 items). Examples of item themes are: Lack of power and influence, management or supervision of other people’s work, having too many things to do. We re-phrased some of the OSI items to reflect direct statements. The response scale ranged from 1 (almost never) to 6 (almost always). Coefficients alpha assessment of scale reliability was 0.80.
• Individual consequences of stress – sub scales: Psychological (PSYTH: 18 items) and physical (PHIT: 12 items) health; both deriving individual scores. Examples of PSYTH items are: During the day, there are moments when you feel worried, upset, useful, confident. We re-phrased some of items to reflect direct statements. Examples of PHIT items are: Did you notice any symptoms over the last 6 months, like lack of appetite, or headache, or noise. The response scale ranged from 1 (almost never) to 6 (almost always). Coefficient alphas were 0.79 for the PSYTH sub-scale and 0.85 for the PHIT sub-scale.

MAAS – Mindfulness Attention Awareness Scale (Brown and Ryan, 2003; Italian version Rabitti et al., 2013) is a 15-item scale designed to assess receptive awareness of and attention to what is taking place in the present moment. Respondents are asked to rate how frequently they experience what is described in each statement using a 6-point Likert scale from 1 (almost always) to 6 (almost never), where high scores reflect a more mindful presence. An example of an item includes “I find it difficult to stay focused on what is happening in the present.” The coefficient alpha for the scale was 0.81.

AAQ. II – The Acceptance and Action Questionnaire - II (Hayes et al., 2006; Bond et al., 2011, Italian version Pennato et al., 2013) focuses on individual willingness to experience unwanted private events, the ability to be in the present moment and the commitment to flexible value-directed actions while experiencing negative internal events. The 10-item version was used in this study. Each item is rated on a 7-point scale ranging from 1 (never true) to 7 (always true), with higher scores indicating greater levels of psychological inflexibility. An example item example is, my thoughts and feelings do not get in the way of how I want to live my life. We re-phrased three of the 10 AAQ. II items to reflect direct statements. Coefficient alpha for the AAQ was 0.74.

### Socio-Demographic Variables

Participants were also asked to provide information on socio-demographic characteristics, such as gender (a dummy variable, 1 = male and 2 = female), age, school grade, and occupational history that may be associated with psychological stress, including: type of contract (such as long-term or fixed-term contract), time of contract (full or part time), work position, shift work, years of service, hours of service for day (Table 1).

Before testing our hypotheses confirmatory factor analysis (CFA) and descriptive analyses were computed. CFA was conducted to examine the factorial structure of the measures used in this study through maximum likelihood estimation. Descriptive analyses (mean, standard deviation, and Bravais–Pearson’s correlation coefficients) were computed to pre-investigate the data as shown in Table 2. An Anova was performed to test subsamples differences (nurses and other healthcare professionals) in dependent scales’ scores; significance was verified for *p* < 0.05. Hypothesis testing (main effects and moderation) was conducted using Structural Equation Modeling (SEM). Data analysis was performed by using SPSS version 22.0, including the AMOS statistical package.

**Table 2:** Study variables: Descriptive statistics and bivariate correlations (*N* = 411).

	*M*	*SD*	1	2	3	4	5	6	7	8	9	10	11	12	13
1. Age	49.16	8.65	–												
2. Gender (1 = M, 2 = F)	–	–	-0.122^*^	–											
3. Area expert	–	–	0.080	0.036	–										
4. Years	15.4	8.63	0.528^*^	-0.058	0.080	–									
5. Work Position	–	–	-0.089	0.150^**^	-0.045	-0.188^**^	–								
6. Man. Decision	4.01	1.09	0.006	0.108^*^	0.000	-0.026	-0.001	–							
7. Man. Power	4.08	1.04	-0.068	0.061	0.044	-0.038	0.039	0.602^**^	–						
8. Relationships	3.92	1.05	-0.033	0.083	0.057	0.001	0.032	0.775^**^	0.735^**^	–					
9. Intrinsic factors	3.57	0.94	0.004	0.077	0.053	0.014	0.027	0.726^**^	0.777^**^	0.800^**^	–				
10. Mindfulness	4.52	0.69	-0.037	0.025	-0.049	-0.061	-0.094	-0.117^*^	-0.169^**^	-0.150^**^	-0.266^**^	–			
11. Flexibility	2.50	1.18	0.27	-0.004	0.091	0.080	0.019	0.044	0.064	0.057	0.061	-0.008	–		
12. Psy. Health	2.81	0.96	0.017	0.153^**^	0.044	-0.012	-0.001	0.295^**^	0.388^**^	0.363^**^	0.458^**^	-0.362^**^	0.81	–	
13. Phy. Health	2.49	1.02	0.047	0.234^**^	0.047	0.111^*^	0.030	0.360^**^	0.359^**^	0.397^**^	0.461^**^	-0.321^**^	0.047	0.531^**^	1

*For Gender, Area of expertise and Work Position, means (M) and standard deviations (SD) are not reported because these variables were categorical in the questionnaire. ^∗^p < 0.05. ^∗∗^p < 0.01.*

## Results

### Confirmatory Factor Analyses

Confirmatory factor analyses affirmed that seven construct measures could be treated as unidimensional, and one as bidimensional with fit indices satisfying standard criteria (e.g., Comparative Fit Index (CFI) >0.90, Bentler, 1990). Root mean square error of approximation (RMSEA) was <0.08 (Hu and Bentler, 1999).

Confirmatory factors analyses results are reported below:

#### Intrinsic Factors (JF)

CFA supported the one factor structure (χ^2^ = 35.531, *df* = 19, *p* = 0.012, χ^2^*/df* = 1.870, CFI = 980, TLI = 0.970, SRMR = 0.034, and RMSEA = 0.046), when the covariance error between two pairs of similar items in the same factor was specified. The composite reliability was 0.80.

#### Relationships (RF)

CFA supported the one factor structure (χ^2^ = 7.779, *df* = 5, *p* = 0.168, χ^2^*/df* = 1.559, CFI = 0.996, TLI = 0.989, SRMR = 0.024, and RMSEA = 0.037), when the error covariance between four pairs of similar items in the same factor was specified. The composite reliability was 0.79.

#### Managerial Factors (MF) (Power and Decision)

CFA supported the two factors structure (χ^2^ = 36.754, *p* = 0.079, *df* = 26, χ^2^/*df* = 1.414, CFI = 990, TLI = 0.986, SRMR = 0.029, and RMSEA = 0.032), when the latent factors were allowed to covary. The two managerial power and decision factors were, respectively, constituted by five and four items. The value of the composite reliability was 0.78 for managerial power and 0.74 for managerial decision.

#### Mindfulness

CFA supported the one factor structure (χ^2^ = 20.834, *p* = 0.288, *df* = 18, χ^2^/*df* = 1.157, CFI = 997, TLI = 995, SRMR = 0.025, and RMSEA = 0.020). The composite reliability was 0.81.

#### Flexibility

CFA supported the one factor structure (χ^2^ = 5.354, *p* = 0.499, *df* = 6, χ^2^/*df* = 0.892, CFI = 1, TLI = 1, SRMR = 0.015, and RMSEA = 0.000). The composite reliability was 0.74.

#### Psychological Health (PSYTH)

CFA supported the one factor structure (χ^2^ = 8.153, *p* = 0.833, *df* = 13, χ^2^/*df* = 0.627, CFI = 1, TLI = 1, SRMR = 0.018, and RMSEA = 0.000). The composite reliability was 0.79.

#### Physical Health (PHIT)

CFA supported the one factor structure (χ^2^ = 54.447, *p* = 0.078, *df* = 41, χ^2^/*df* = 1.328, CFI = 99, TLI = 99, SRMR = 0.027, and RMSEA = 0.028). The composite reliability was 0.85.

### Descriptive Statistics

On the basis of the CFA results, aggregates of the selected items were computed for the following analyses (including, bivariate correlations and multivariate analysis).

Means (M), standard deviations (SDs), and bivariate correlations for the measured variables are presented in Table 2.

The Bravais–Pearson’s *r* correlation coefficients between socio-demographic indexes and the psychological variables were statistically significant for: gender and managerial decision, psychological health, physical health, gender, years of experience in organization, and physical health.

With regard to the correlations among the psychological constructs: managerial decision, managerial power, relationships, and intrinsic factors showed a significant positive correlation with psychological and physical health; managerial decision, managerial power, relationships, and intrinsic factors were significantly and positively correlated with each other and negatively correlated with mindfulness. Flexibility was not correlated with any psychological constructs.

The correlation between socio-demographic indexes (gender, age, years, area of expertise, and work position) and social-psychological variables was significant (and positive) only for managerial factor (decision and power) and research unit. Managerial factor was negatively correlated with gender. Research unit was negatively correlated with intrinsic factor of work (JF). All other relationships with socio-demographic variables were not significant.

Inter-correlations among OSI variables (MF; RF; and JF), showed a significant positive relationship with mindfulness and both dimensions of health, but not with flexibility. With respect to mindfulness interaction, results showed a significant positive relationship on all two dimensions of health (psychological and physical).

Correlations among such aggregates were calculated in order to examine discriminant validity, determining that all constructs in the model are distinct from each other. All the correlations were below the threshold of 0.80 as suggested by Quintal et al. (2010).

### Hypothesis Test

In order to explore the research hypotheses on the causal relationships among variables, we used structural equation modeling. Before testing SEM, we performed an Anova test between two different samples (nurses and other healthcare professionals) that could have different patterns in physical and psychological health. Then, we tested three alternative models because with the SEM we can only evaluate the degree to which a measurement or path model is consistent with data. A perfect fit is usually trivial because the model includes all possible paths between all pairs of variables (e.g., Boomsma, 2000; Steiger, 2001). As the authors suggest, the best way for researchers to address this is to present the proposed model as well as one or more theoretically plausible models representing competing hypotheses. We estimated the parameters of two of the three models (1 and 2) with maximum likelihood estimation. For the third model (Mindfulness Moderation Effect) we used the asymptotical distribution that is robust to the violation of the normality assumptions (Yuan and Bentler, 2005). The Mindfulness variables presented values of asymmetry and kurtosis over -1.5 and +1.5 that are not considered acceptable in order to prove normal distribution (Tabachnick and Fidell, 2013).

#### Anova Test

An Anova was performed to test subsamples differences in dependent scales’ scores; significance was verified for *p* < 0.05. No statistical differences emerged for Psychological and Physical Health in the two sample groups (nurses and other healthcare professionals; *p* > 0.05).

##### Model 1

The first model included the main effects of independent variables (MF both decision and power, relationships, and intrinsic factors on psychological and physical health (H1, H2, H3, and H4).

##### Model 2

The second model considered the moderating effect of flexibility on the relationship between each independent variable (managerial decision, managerial power, relationship, and intrinsic factors) on psychological and physical health (H5, H6, H7, and H8).

##### Model 3

The last model considered the moderating effect of mindfulness on the relationship between each independent variable (managerial decision, managerial power, relationship, and intrinsic factors) on psychological and physical health (H9, H10, H11, and H12).

#### Model 1 (Main Effects of Independent Variables on Psychological and Physical Health)

Positive relationships emerged between the following variables: (i) Intrinsic factors both on psychological (β = 0.458, *p* < 0.001) and physical health (*β* = 0.461, *p* < 0.001); (ii) Relationship both on psychological (β = 0.363, *p* < 001) and physical health (β = 0.397, *p* < 0.001); (iii) Managerial decision both on psychological (β = 0.295, *p* < 0.001) and physical health (β = 0.360, *p* < 0.001) and; (iv) Managerial power on both psychological (β = 0.388, *p* < 0.001) and physical health (β = 0.359, *p* < 0.001).

Thus H1, H2, H3, and H4 are partially supported.

The overall fit of the structural model was, respectively as follows:

χ^2^ = 73.670, *df* = 1, *p* = 0.000, χ^2^/*df* = 93.824, TLI = 0.177, CFI = 0.726, SRMR = 0.070, and RMSEA = 0.130;
χ^2^ = 93.824, *df* = 1, *p* = 0.000, χ^2^/*df* = 93.824, TLI = 0.270, CFI = 0.577, SRMR = 0.070, and RMSEA = 0.157;
χ^2^ = 105.608, *df* = 1, *p* = 0.000, χ^2^/*df* = 105.608, TLI = 0.595, CFI = 0.468, SRMR = 0.173, and RMSEA = 0.505;
χ^2^ = 95.268, *df* = 1, *p* = 0.000, χ^2^/*df* = 105.608, TLI = 0.311, CFI = 0.563, SRMR = 0.160, and RMSEA = 0.480;

In contrast to the test of the main effect of IV on DV (*p* < 001), the overall model fit does not provide evidence of an acceptable model. The goodness of fit was evaluated on the basis of the criteria specified from different authors. Such indices are the Tucker Lewis Index (TLI; Tucker and Lewis, 1973), which should be close to 0.95; the CFI; (Bentler, 1990), which should be close to 0.95; the RMSEA, which should have values close to 0.06, with values around 0.08 representing reasonable errors of approximation, according to Browne and Cudeck (1993); and the Standardized Root Mean Residual (SRMR), which should have values less than 0.05 (Diamantopoulos and Siguaw, 2000), however, values not higher than 0.08 are deemed acceptable (Hu and Bentler, 1999).

#### Model 2 (Flexibility Moderation Effect)

These models examined the moderating role of flexibility on the relationship between each independent variable (MF both decision and power, relationships, and intrinsic factors) on psychological and physical health. In order to run these models, an interaction term was computed in the model between flexibility and each independent variable, by calculating the multiplication of the standardized variables.

The results did not show a significant effect of the interactive term on flexibility (*p* < 0.05), thus H5, H6, H7, and H8 were not supported.

#### Model 3 (Mindfulness Moderation Effect)

The following models examined the moderation role of mindfulness on the relationship between each independent variable: MF both decision and power; relationship, and intrinsic factors on psychological and physical health. In order to run this model, an interaction term was computed in the model between mindfulness and each independent variable, by calculating the multiplication of the standardized variables.

For higher levels of mindfulness only a moderation effect emerged for the following relationships: (i) Managerial decision on psychological health (managerial decision X mindfulness: β = -0.090, *p* < 0.05); (ii) Relationship on psychological health (relationship X mindfulness: β = -0.100, *p* < 0.05). For lower levels of mindfulness, the relationship between managerial decision on psychological health and the relationship between psychological health and relationship (β = 0.324, *p* < 0.001) is positive and significant (β = 0.266, *p* < 0.001).

**FIGURE 2:**
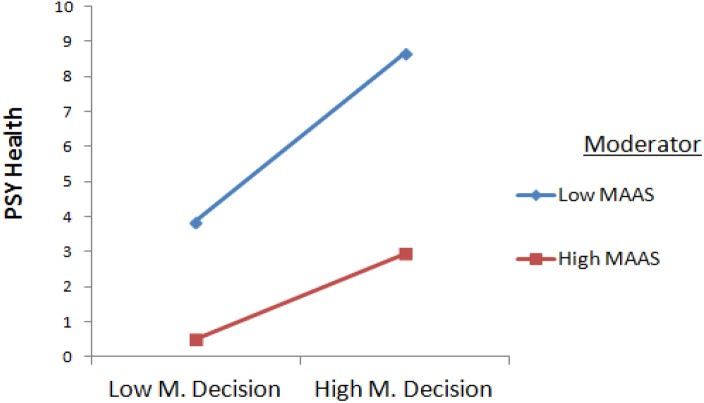
Interaction Between Managerial Decision and Mindfulness.

Higher managerial decision was lead to higher psychological health for low mindfulness values, but was not associated with higher psychological health for higher MAAS (Figure 1, 2).

**FIGURE 3:**
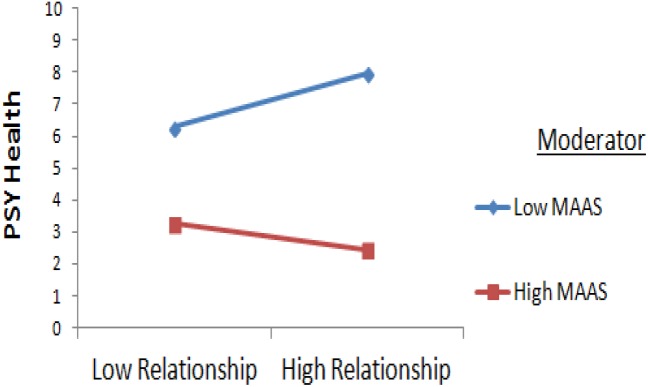
Interaction Between Relationship and Mindfulness.

Higher scores on relationships was associated with higher psychological health for low MAAS values, but was not associated with higher psychological health in higher mindfulness (Figure 2, 3).

These results were confirmed by a good overall fit of the structural model, as evidenced by indices for the first model (χ^2^ = 0.000, *df* = 0, *p* = 0, χ^2^/*df* = 0.0, TLI = 1, CFI = 1, SRMR = 0.000, and RMSEA = 0.200) and for the second one (χ^2^ = 0.000, *df* = 2, *p* = 000, χ^2^/*df* = 0, TLI = 0.1, CFI = 0.1, SRMR = 0.000, and RMSEA = 0.020).

The results showed a significant effect of the interactive term on mindfulness; thus, H9, H10, H11, and H12 were supported.

Overall the assessment of fit indices led to the choice of Model 3, which includes the moderating effect of mindfulness on the relationship between managerial decision and psychological health and relationships and psychological health.

## Discussion and Limitations

This study aimed to explore the relationships between occupational stress factors and their consequences across the health pathway in workers of an Italian hospital. Furthermore, the moderating effect of psychological flexibility (Bond and Flaxman, 2006) and mindfulness (Bond and Bunce, 2003), on psychological and physical health was investigated. In this study three alternative models were tested. Authors (e.g., Boomsma, 2000; Steiger, 2001) suggest testing one or more theoretically plausible models representing competing hypotheses. These alternative models helped us to choose the best model with the better fit. Further, because physical and psychological health at work, could have different patterns from nurses and other healthcare professionals (Tomietto et al., 2019), we performed Anova test on the different subsamples.

The first one tested the hypothesis that MF, both decision and power, relationships and intrinsic factors are negatively correlated with psychological and Physical Health (H1; H2; H3; and H4), where they may be associated with more positive psychological adjustment to stress. Our data partially supports this hypothesis, demonstrating a positive and significant correlation between the effect of independent variables (MF both decision and power, relationships and the two outcomes assessed, psychological and physical health).

The second model tested the moderating effect of psychological flexibility on the relationship between MF (power and decision), relationships, intrinsic factors, and psychological and physical health (H5; H6; H7; and H8). In this case, our data did not support this hypothesis and showed no significant effect.

The last and third hypothesis that mindfulness moderates the relationship between each independent variable (managerial decision, managerial power, relationship at work and intrinsic factors) on psychological and physical health (H9; H10; H11; and H12). Our data supported this hypothesis, demonstrating a significant effect of the interactive term on mindfulness.

Mindfulness is one component of the ACT model related to the left and central processes in the so called hexaflex of psychological flexibility. The second tested model did not offer support for the moderating role of psychological flexibility, while the third model supported the moderating role of mindfulness. This might be viewed as contradictory though it could be explained by several factors. One could be relevant to the 10-item version of the scale, used in the study, which potentially has some drawbacks that led the authors to eliminate three of them in its latest version (Bond et al., 2011). In addition, psychological flexibility is context dependent. For example, workers can show patterns of behavioral flexibility with their significant other in their relationship while being less flexible in the workplace. This means that other variables that are relatively stable across contexts may be more strongly correlated with the AAQ-II, while work related variables may not (Bond et al., 2013). This is to say that when using the AAQ-II subjects are requested to rate flexibility in the broader context of their life, which include not only the above-mentioned mindfulness processes but also commitment in a more general valued path of actions. This more general context can be unrelated to the factors considered in the second model and thus bringing no evidence of a moderating role for PF on MF and psychological health. And yet when mindfulness per se is considered, this process may still have a mediating role.

Further, mindfulness showed a high level in this in this study and specific context (*M* = 3.99 on a scale from 1 to 6), while flexibility was low (*M* = 2.50 on a scale from 1 to 7). So, mindfulness varied enough to play a role as a buffering variable, at least on psychological health. Furthermore, in this context no difference between subsamples were detected. All workers in the hospital perceived a low level of psychological (*M* = 2.81 on a scale from 1 to 6) and physical health (*M* = 2.49 on a scale from 1 to 6) and those, presumably, is given by a relatively good organizational climate.

To overcome those issues related to the AAQ-II as a broader measure of flexibility, Bond et al. (2013) developed the Work-Related Acceptance and Action Questionnaire (WAAQ) to measure work environment related flexibility, which was recently used to evaluate psychological flexibility of the nursing staff of a cancer hospital (Xu et al., 2018). Results suggest that the WAAQ is a better tool to measure psychological flexibility in the workplace. Unfortunately, no Italian validated version of this scale was available at the time when this research was planned.

To date, research on psychological flexibility and ACT in workplaces has not been systematically conducted in Italy. Our study provides further evidence that psychological flexibility is a transcultural construct (Monestès et al., 2016) and that the association between psychology flexibility and outcome measures is independent of the effects of any demographic variables that were assessed. In this study the correlation between socio-demographic indexes (gender, age, years, area of expertise and work position) and social-psychological variables was significant (and positive) only for managerial factor, and negative only for gender. Research unit was negatively correlated with intrinsic factor of work. All other relationships between socio-demographic variables were not significant. It was important to control these variables to explore the potential for psychological adaptation being this a somewhat independent process, occurring naturally over time, or in response primarily to life experiences. In other words, this natural context, where psychological flexibility was not specifically trained and assessed as a pattern of behaviors that might be acquired via personal history, demonstrates the importance of acceptance of all internal events.

In line with literature, overall our data shows the emergence of an association between all the occupational stress factors and individual and organizational symptoms of occupational ill-health in hospitals. Leigh et al. (2000) stressed the importance of assessing and analyzing the foundation of interventions by identifying the kinds of HRD specific to the organization. Our findings also suggest that interventions aimed at targeting mindfulness processes may also have a more general effect, including quality of life, in line with the broader ACT literature (Longmore and Worrell, 2007). In the specific case of health workers, the contact with patients is an emotionally stressing context that can threaten wellbeing outcomes and impact work performance. An intervention with effects on overall psychological wellbeing, might be more useful in encouraging a more adaptive approach to stress treatment and its consequences across the health pathway (Mischel, 1969; Miller et al., 1999; Runco, 2007; Platania et al., 2015). Though our data are observational and cross-sectional only, making it difficult to draw conclusions on causality, they are nevertheless a useful baseline and rationale for the development of mindfulness-based interventions that may reduce distress and psychological comorbidity.

ACT-based interventions, for example, found, enhanced psychological flexibility with a beneficial impact in improving job control (Bond et al., 2008; Moran, 2015). By promoting a mindful attitude people in the intervention group were better at noticing where, when and the degree to which they had increased levels of control. ACT protocols have demonstrated improvement in employees’ mental health (Bond and Bunce, 2000; Flaxman and Bond, 2010; Bond et al., 2016), enhancement of their ability to be innovative (Bond and Bunce, 2000), and a reduction in emotional burnout (Lloyd et al., 2013; Waters et al., 2018). These interventions can be successfully conducted also in the public administration settings (Macías et al., 2019), a state-run context similar to the one of the health workers of the Italian National Health System, interviewed for this research.

Mindfulness-base ACT protocols have also been shown to be effective in preventing stress-related health issues. Dahl et al. (2004) offered an ACT intervention to a group of Swedish care workers at high risk of long-term work disability due to stress and musculoskeletal pain. The control group received their respective medical treatment as usual (MTAU). At the end of the intervention and at a 6-months follow-up, participants in the ACT group showed fewer sick leave days and used fewer drugs than those in the control condition, with a mean reduction of 90% in sick day leave. Acting on psychological flexibility could be the key to stress reduction and health prevention in the workplace, where short intervention programs can have positive effects (Boyatzis and McKee, 2013).

Acceptance and Commitment Therapy (Hayes et al., 1999) is one of the most effective models of intervention to undermine dysfunctional problematic patterns linked to the verbal processes of experiential avoidance and to help build more functional and adaptive value-oriented repertoires. Though the emphasis on goal-directed behavior is not unique in psychology literature (e.g., Bandura, 1986; Klein, 1989; Locke and Latham, 1990) mindfulness strengthens the relation between people’s action and how psychologically flexible they are. In addition, by getting in touch with one’s internal experiences, people are not expending time and cognitive resources on the task of trying to control and down-regulate their psychological experiences. Consequently, they have more resources to notice goal-related and contingency-driven opportunities that exist in their current situation.

There are some limitations to this study. Generally, it provides partial support to the research hypotheses. Limitations need to be better addressed in future research by using tools such as the WAAQ or the 7-item AAQ to better investigate work-specific and more general aspects of psychological flexibility. Results should be interpreted with caution because of the use of a cross-sectional survey which is not sufficient for establishing a causal relationship. Another limitation of this study was the sampling of work positions with response rates being lower than ideal. This was owing to service contingencies and being an emergency hospital, where not all wards had the available time to take part in the survey. Results may have been affected by specific features of the investigated unit. This research aims to include those departments which were not part of this study. For more complex designs, including intervention studies and cohort designs, larger samples would be required and would allow more sophisticated statistical analysis. We would also like to draw attention to the fact that our sample did not include ethnic and cultural diversity. Our work is not unique in this, but it is an important consideration for future research, particularly in the development of interventions which may be more prone to participant effects, and in which cultural variation may impact patient-perceived acceptability and effectiveness. Thus, for all these limitations, experimental studies are particularly needed.

## Author Contributions

TR and GP conceived and designed the experiments. DB analyzed the data and helped the co-authors conceive and design the research. TR contributed the analysis tools. GS critically reviewed the manuscript. TR wrote the manuscript.

## Conflict of Interest Statement

The authors declare that the research was conducted in the absence of any commercial or financial relationships that could be construed as a potential conflict of interest.

## Footnotes

1 https://www.ispettorato.gov.it/it-it/Documenti-Norme/Documents/Testo-Unico-Dlgs-81-08-edizione-di-luglio-2018.pdf

2 https://www.eurofound.europa.eu/publications/article/2005/work-related-stress
